# Bismuth-Based Ceramic Processed at Ultra-Low-Temperature for Dielectric Applications

**DOI:** 10.3390/nano16010046

**Published:** 2025-12-29

**Authors:** Susana Devesa, Sílvia Soreto Teixeira, Manuel Pedro Graça, Luís Cadillon Costa

**Affiliations:** 1 Department of Mechanical Engineering, CEMMPRE, ARISE, University of Coimbra, Rua Luís Reis Santos, 3030-788 Coimbra, Portugal; 2 i3N and Department of Physics, University of Aveiro, Campus Universitário de Santiago, 3810-193 Aveiro, Portugal; silvia.soreto@ua.pt (S.S.T.); mpfg@ua.pt (M.P.G.); kady@dem.uc.pt (L.C.C.)

**Keywords:** Bi–Fe–Nb oxide system, ultra-low-temperature processing, dielectric properties, impedance spectroscopy

## Abstract

High-performance dielectric materials that can be processed at ultra-low temperatures are essential for next-generation LTCC technologies and compact RF–microwave components. In this work, a multicomponent Bi–Fe–Nb oxide system was synthesized using a modified citrate sol–gel method and thermally treated at only 400 °C to investigate its structural evolution and dielectric behavior. XRD and Raman analysis revealed the coexistence of a well-crystallized BiOCl phase embedded within a partially amorphous Bi–Fe–Nb–O matrix. SEM and EDS mapping confirmed the presence of two distinct microstructural regions, reflecting differences in local composition and crystallization kinetics. Microwave measurements at 2.7 and 5.0 GHz showed low dielectric losses and a stable dielectric response. Impedance spectroscopy in the RF range revealed strong Maxwell–Wagner polarization at low frequencies and thermally activated relaxation evidenced by the temperature shift in the modulus and impedance peaks. Arrhenius analysis of the relaxation frequencies yielded similar activation energies from both modulus and impedance formalisms, indicating a single underlying relaxation mechanism. Equivalent-circuit fitting confirmed non-Debye behavior, with nearly temperature-independent capacitance and decreasing resistance consistent with thermally activated conduction. These results demonstrate that the Bi–Fe–Nb system exhibits promising dielectric stability and functional behavior even when processed at exceptionally low temperatures.

## 1. Introduction

Recent and rapid developments in wireless telecommunications, the Internet of Things (IoT), the Tactile Internet (5G networks), the Industrial Internet, electronic warfare, satellite communications, and intelligent transportation systems have created a strong demand for low-loss dielectric materials that can be sintered at ultra-low temperatures. Consequently, extensive research efforts are now focused on discovering materials with firing temperatures below 700 °C, leading to a rapid increase in publications on this topic [1,2,3,4,5,6,7].

Given that the use of low-temperature co-fired ceramics (LTCCs) has been demonstrated to be one of the most effective approaches for minimizing device size and circuit area, this research topic continues to be of significant relevance [8].

Currently, four main approaches are employed to develop low-sintering-temperature materials for LTCC devices. One approach involves using glass-ceramics or glass–ceramic composites as the matrix, with their sinterability and dielectric properties tailored through suitable additives. Another strategy focuses on dielectric material systems with inherently low sintering temperatures, such as MoO_3_-, TeO_2_-, P_2_O_5_-, WO_3_-, Bi_2_O_3_-, and V_2_O_5_-based compounds. A further method employs chemically synthesized nanopowders as raw materials to reduce the temperature required for ceramic densification. And, finally, sintering aids or low-melting-point oxides can be incorporated into ceramics to further lower the sintering temperature [8,9,10].

However, each of these approaches has limitations. Glass-ceramics and glass–ceramic composites may suffer from composition volatilization, unstable dielectric performance, and reactions with metal electrodes, leading to the decline of the dielectric properties [8].

Low-sintering-temperature dielectric systems face challenges such as high cost (TeO_2_) [8], toxicity of raw materials (V_2_O_5_ and WO_3_) [11], and large temperature coefficients, limiting their application in LTCC devices [8]. Chemically synthesized nanopowders, while effective, are costly and time-consuming to produce. Sintering aids and low-melting-point oxides, though widely used, require careful optimization to avoid negatively affecting the material performance [8].

In this study, a multicomponent Bi–Fe–Nb oxide system was explored as a promising ultra-low-temperature processed material. Unlike conventional strategies that focus on obtaining fully crystallized, single-phase oxides, this approach deliberately targets the intermediate phases and structural evolution of the precursor matrix under mild thermal conditions.

By investigating these partially crystallized states, the method provides unique insight into phase transformations, decomposition pathways, and precursor dynamics that are typically overlooked in traditional low-sintering/processing approaches, which prioritize high crystallinity. This focus on intermediate products not only reveals the potential of multicomponent Bi–Fe–Nb systems but also offers a pathway to tailor functional properties, such as dielectric behavior and sinterability, through controlled thermal processing.

In materials science, the composition, structure, performance, and application of materials are intricately interconnected. Among these relationships, composition design and ceramic processing play particularly crucial roles. Within ceramic processing, sintering/processing parameters are especially significant, as factors such as phase structure, point defects, grain size, and density are highly sensitive to both the sintering/processing temperature and the holding time, effects that are especially pronounced in bismuth-containing ceramics [12].

Among the dielectric materials, LTCCs have attracted significant attention for multilayer radiofrequency and microwave components, with bismuth-containing oxides standing out due to their structural versatility and promising functional properties, which make them suitable for applications in catalysis, optics, nanophotonics, and nanoelectronics [13]. Taking these factors into consideration, the Bi–Fe–Nb oxide system was chosen for the present study owing to its favorable synthesis conditions and encouraging preliminary experimental findings [14,15,16].

## 2. Materials and Methods

### 2.1. Synthesis of the Bi-Based System

A multicomponent oxide system containing Bi, Fe, and Nb was prepared using a modified citrate sol–gel method. Bismuth(III) nitrate pentahydrate (Bi(NO_3_)_3_·5H_2_O, 98%, Sigma-Aldrich), niobium(V) chloride (NbCl_5_, 99%, Merck, Darmstadt, Germany), and iron(III) nitrate nonahydrate (Fe(NO_3_)_3_·9H_2_O, 98%, Mateck, Nordrhein-Westfalen, Germany) were used as starting materials in a molar ratio of 0.75:1.00:0.25. These precursors were dissolved in a minimal volume of 3% (*v*/*v*) hydrogen peroxide (H_2_O_2_, Sigma-Aldrich, St. Louis, MO, USA) to aid dissolution and minimize hydrolysis side reactions.

Citric acid (C_6_H_8_O_7_, 99%, Sigma-Aldrich), at a 1:1 molar ratio with the total metal ions, and ethylene glycol (C_2_H_6_O_2_, 99%, Sigma-Aldrich) were added as the chelating agent and polymerization medium, respectively. The mixture was stirred continuously for 168 h at room temperature to promote homogeneity and increase viscosity. The resulting gel was dried at 300 °C for 60 h to remove solvents and initiate decomposition of the organic content.

The dried powder was then ground and isostatically pressed into circular pellets (6.20 mm in diameter and 1.25 mm in height) and cylinders (3.00 mm in diameter and 5.75 mm in height) using a hydraulic press. These were thermally treated at 400 °C for 4 h in air at a heating rate of 5 °C/min, in accordance with our previous work [15].

### 2.2. Characterization Techniques

Structural characterization was carried out by X-ray diffraction (XRD) using an Empyrean diffractometer (CuKα radiation, λ = 1.54060 Å) operated at 45 kV and 40 mA, in a Bragg–Brentano parafocusing configuration. Data were collected using a step size of 0.02° and a counting time of 1 s per step, over a 2θ range of 10–60°.

Raman spectroscopy was performed with a Renishaw inVia™ confocal Raman spectrometer (Wotton-under-Edge, UK) using a 532 nm green laser.

The morphology was examined by scanning electron microscopy (SEM) with a Hitachi SU3800 microscope (Hitachi, Tokyo, Japan), in both secondary electron and backscattered electron modes.

Elemental composition was analyzed by energy-dispersive X-ray spectroscopy (EDS) using a Bruker Nano system.

Dynamic Light Scattering (DLS) measurements were performed using a ZS XPLORER instrument (Malvern Panalytical, Malvern, Worcestershire, UK) equipped with a DTS0012 cell and operating at 25 °C. Samples were dispersed in ultrapure water (refractive index: 1.33; viscosity: 0.887 cP) and analyzed under steady-state conditions. Each measurement yielded the Z-Average hydrodynamic diameter and the volume-weighted particle size distribution, as well as the statistical contribution of individual populations (peaks). The analysis was conducted using the ZS XPLORER software (version 4.1.0.82), and for each measurement, three sequential scans were acquired to ensure stability and reproducibility of the signal.

In the microwave range, the measurements were performed at room temperature using the small perturbation method on cylindrical samples, with two cavities operating in the T_E01n_ mode at resonant frequencies of 2.7 and 5.0 GHz, coupled to an HP 8753D Network Analyzer (Santa Clara, CA, USA).

In the radiofrequency range, the electrical characterization was carried out by impedance spectroscopy using a precision impedance analyzer (Agilent 4294A, Santa Clara, CA, USA) in the Cp–Rp configuration, with the temperature varying between 200 and 330 K and the frequency range extended from 100 Hz to 1 MHz. Before the measurements, which were performed in a helium atmosphere to eliminate moisture and improve heat transfer, silver electrodes were applied to the surfaces of the pellet using silver ink.

## 3. Results

### 3.1. Structural Characterization

**Figure 1:**
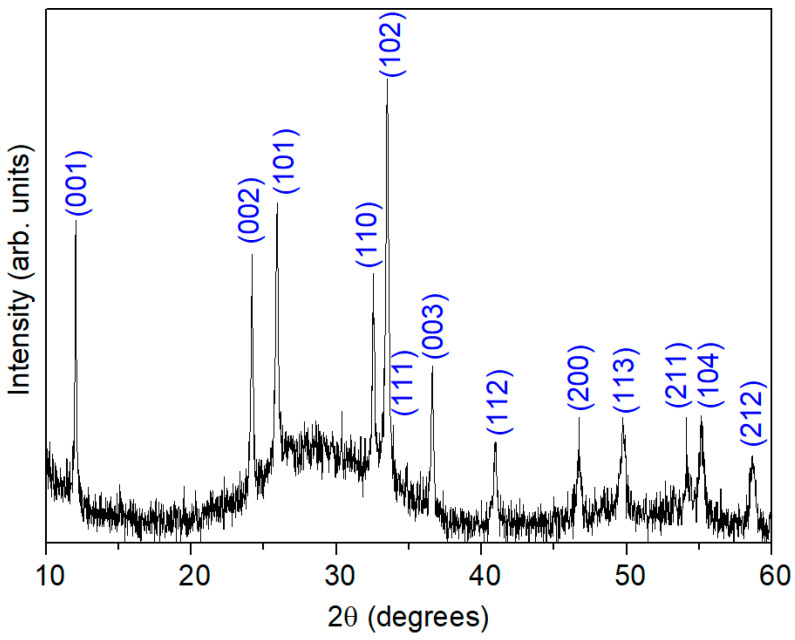
X-ray diffraction (XRD) pattern of the Bi-based system thermally treated at 400 °C. The labeled Miller indices correspond to the crystalline BiOCl phase (COD 1011175), while the broad low-angle hump indicates the presence of a partially amorphous Bi–Fe–Nb–O matrix.

Figure 1 shows the XRD diffractogram of the Bi-based system. Several well-defined peaks can be observed and indexed to the crystalline phase BiClO. The Miller indices, also depicted, correspond to those reported in COD 1011175 [17]. Additionally, a broad hump appears in the low-angle region of the pattern, which is not indexed to the BiClO phase. This amorphous fraction is expected to contain iron (Fe) and niobium (Nb) as essential components, likely together with bismuth and oxygen, forming a complex oxide. One possible composition is Bi_1.34_Fe_0.66_Nb_1.34_O_6.35_, which has its main peak at 24.9° [14], consistent with the center of the observed hump. This suggests that part of the sample exists in a poorly crystalline or amorphous state, likely due to incomplete crystallization.

**Figure 2:**
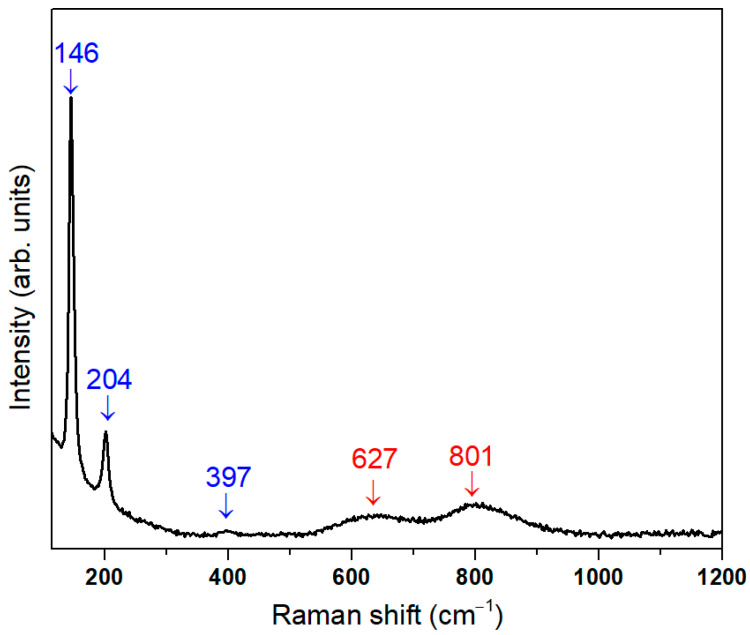
Raman spectrum of the Bi-based system, showing characteristic vibrational modes associated with crystalline BiOCl (sharp bands, identified in blue) and a partially amorphous Bi–Fe–Nb–O phase (broader bands, identified in red), confirming the coexistence of crystalline and amorphous components.

The Raman spectrum of the Bi-based system, presented in Figure 2, exhibits several distinct vibrational bands that can be attributed to both the crystalline BiClO phase and the amorphous Bi_1.34_Fe_0.66_Nb_1.34_O_6.35_ phase.

The bands observed at 146, 204, and 397 cm^−1^ (highlighted in blue) correspond to characteristic vibrational modes of the crystalline BiClO structure. The band at 146 cm^−1^ can be assigned to the symmetrical stretching vibration of the Bi–Cl bond, while the band at 204 cm^−1^ is associated with the symmetric expansion vibration of the Bi–Cl bond. The weak and broad band centered at 397 cm^−1^ can be attributed to oxygen atom vibrations in the BiOCl lattice [18]. These sharp and well-defined peaks indicate the presence of a highly crystalline BiClO fraction in the sample.

The broader bands centered at 627 and 801 cm^−1^ (highlighted in red) are associated with the amorphous phase. Their positions and broad nature are consistent with a poorly crystalline or amorphous Bi_1.34_Fe_0.66_Nb_1.34_O_6.35_ structure [16].

Overall, the Raman spectrum confirms the coexistence of a well-crystallized BiClO phase alongside a partially amorphous Bi_1.34_Fe_0.66_Nb_1.34_O_6.35_ phase, consistent with the XRD observations of a broad low-angle hump corresponding to the amorphous fraction.

### 3.2. Morphological Characterization, Elemental Analysis, and Particle Size Distribution

**Figure 3:**
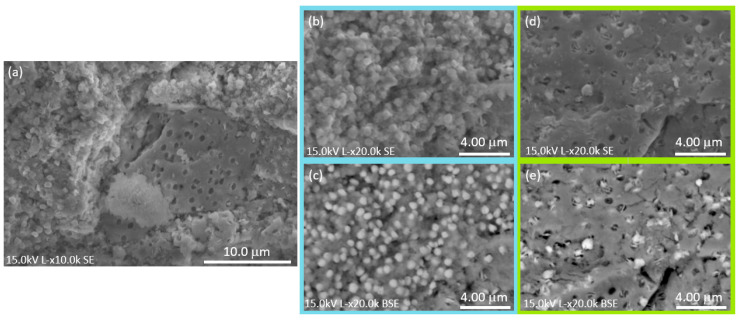
SEM micrographs of the Bi-based system: (**a**) SE image at 10.0 kx showing two distinct morphologies; (**b**) higher-magnification SE image (20.0 kx) of the first morphology; (**c**) corresponding BSE image of (**b**), where brighter regions correspond to crystalline BiClO and darker regions to the amorphous Bi_1.34_Fe_0.66_Nb_1.34_O_6.35_ phase; (**d**) higher-magnification SE image (20.0 kx) of the second morphology; (**e**) corresponding BSE image of (**d**), where brighter regions correspond to crystalline BiClO and darker regions to the amorphous Bi_1.34_Fe_0.66_Nb_1.34_O_6.35_.

The SEM micrograph, Figure 3a, reveals the presence of two distinct morphologies within the Bi-based system. The first morphology (Figure 3b,c) consists of granular agglomerates composed of approximately spherical particles. BSE imaging confirms that this granular region contains a mixture of both phases: the brighter particles correspond to crystalline BiClO, while the darker matrix is associated with the amorphous Bi_1.34_Fe_0.66_Nb_1.34_O_6.35_ phase.

The second morphology (Figure 3d,e) exhibits a more compact, yet porous, structure. BSE contrast indicates that this region is predominantly composed of the amorphous Bi_1.34_Fe_0.66_Nb_1.34_O_6.35_ phase, within which a small fraction of brighter BiClO grains can be observed distributed throughout the matrix.

The coexistence of two distinct morphologies can be attributed to differences in local chemical composition and crystallization kinetics: BiClO tends to crystallize rapidly into granular particles, while the Bi_1.34_Fe_0.66_Nb_1.34_O_6.35_ phase forms more slowly as a compact, porous amorphous matrix, within which BiClO particles may become impregnated [19,20].

**Figure 4:**
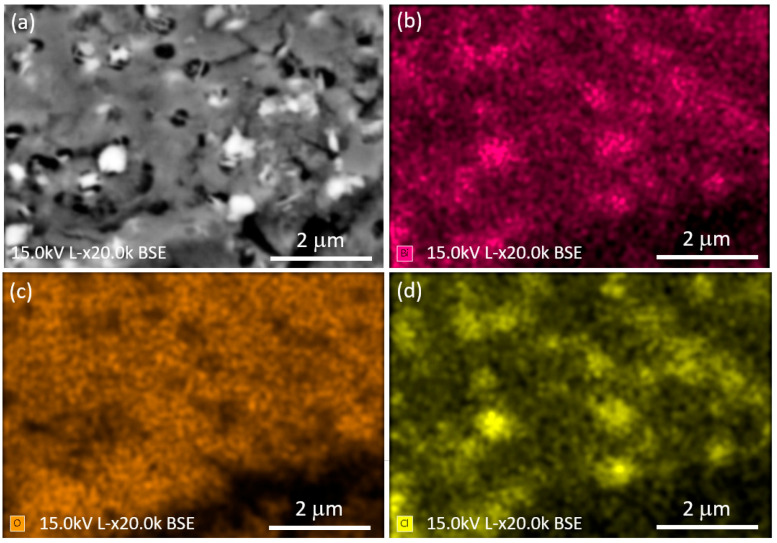
(**a**) Backscattered-electron SEM (BSE–SEM) image of the compact porous region of the Bi-based system; (**b**–**d**) corresponding EDS elemental maps of Bi, O, and Cl, respectively. The elemental distributions highlight BiOCl grains embedded within a Bi–Fe–Nb–O matrix, consistent with phase coexistence inferred from XRD and Raman analyses.

Figure 4a shows the BSE–SEM image of the compact porous region, while Figure 4b–d display the corresponding EDS elemental maps for Bi, O, and Cl, respectively. The EDS elemental maps highlight the presence of BiOCl grains embedded within the matrix, with the distributions of Bi and Cl clearly showing the BiOCl phase, while the slightly lower oxygen signal observed inside the BiOCl grains is consistent with their lower oxygen atomic density compared to the Bi_1.34_Fe_0.66_Nb_1.34_O_6.35_ matrix.

**Figure 5:**
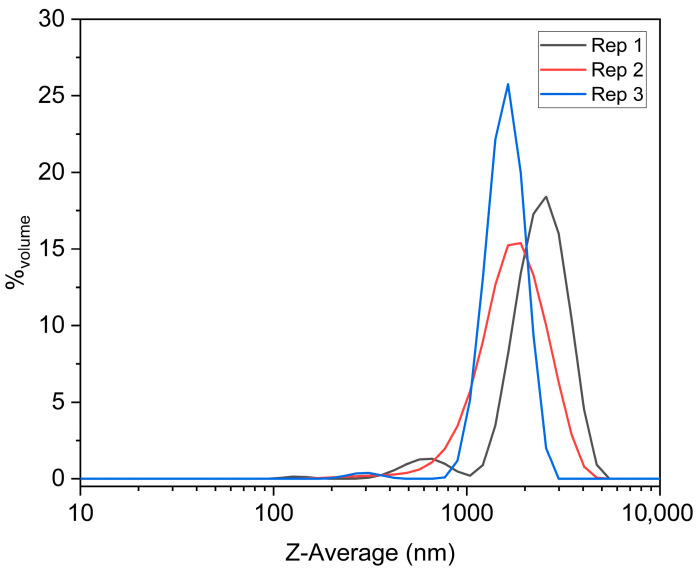
Volume-weighted DLS size distributions measured in triplicate at 25 °C for the Bi-based powders. The sample exhibits a multimodal profile with dominant micrometric aggregates. The Z-Average was 640 ± 7 nm and the PI ranged from 0.394 to 0.416, indicating high polydispersity. Three independent measurements (Rep 1, Rep 2, and Rep 3) are shown, each corresponding to the average of three consecutive runs, and expressed as volume-weighted size distributions (% volume).

The DLS results, presented in Figure 5, revealed a polydisperse profile, with multimodal distributions indicating the presence of significant aggregation, exhibited a hydrodynamic diameter, with a Z-Average of 640 ± 7 nm, accompanied by a polydispersity index (PI) ranging from 0.394 to 0.416 across the three independent measurements, confirming the broad size distribution and limited homogeneity of the dispersion. Two major populations were detected at ~1.36 µm (68%) and ~1.42 µm (47%), along with a residual nanoscale mode at ~135 nm (0.3%), suggesting extensive particle association and limited colloidal stability. This distribution confirms that this system experience substantial aggregation under the tested conditions, with the micrometric modes dominating the volume fraction and overshadowing the contribution of primary nanoparticles.

These results are well aligned with the microstructural heterogeneity revealed by SEM and the partial amorphous–crystalline coexistence observed by XRD and Raman analysis.

### 3.3. Microwave Dielectric Characterization Using the Resonant Cavity

Several techniques can be used to measure the permittivity in the microwave frequency range, including the cavity resonant method. In this approach, the resonance peak frequency and the quality factor of the cavity, with and without the sample, are used to determine the complex dielectric permittivity, ε*, of the material. The shift in the cavity’s resonant frequency, *f*, caused by the insertion of the sample, is related to the real part of the complex permittivity (or dielectric constant), *ε*′. Similarly, the change in the inverse of the quality factor, (*1*/*Q*), provides information on the imaginary part (or dielectric loss), *ε*″. These relations are straightforward when considering only the first-order perturbation in the electric field caused by the sample, according to small perturbation theory, and are presented in Equations (1) and (2). However, to satisfy this condition, the sample size must be much smaller than that of the cavity, and the perturbation must be sufficiently large to be measurable [21,22].

(1) $$\varepsilon^{\prime }=K\frac{f_{0}-f_{l}}{f_{0}}\frac{V}{v}+1$$

(2) $$\varepsilon^{\prime \prime }=\frac{K}{2}\left(\frac{1}{Q_{l}}-\frac{1}{Q_{0}}\right)\frac{V}{v}$$

Here, K is a constant related to the depolarization factor, which depends on the geometric parameters; v is the volume of the sample and V is the volume of the cavity; and the indices 0 and l refer to the empty cavity (in this case, including the sample holder) and the cavity loaded with the sample, respectively. Using a material with a known complex permittivity, the value of K can be determined [23]. In this study, a polytetrafluoroethylene (PTFE) cylinder, with the same size and shape as the samples, was used.

**Figure 6:**
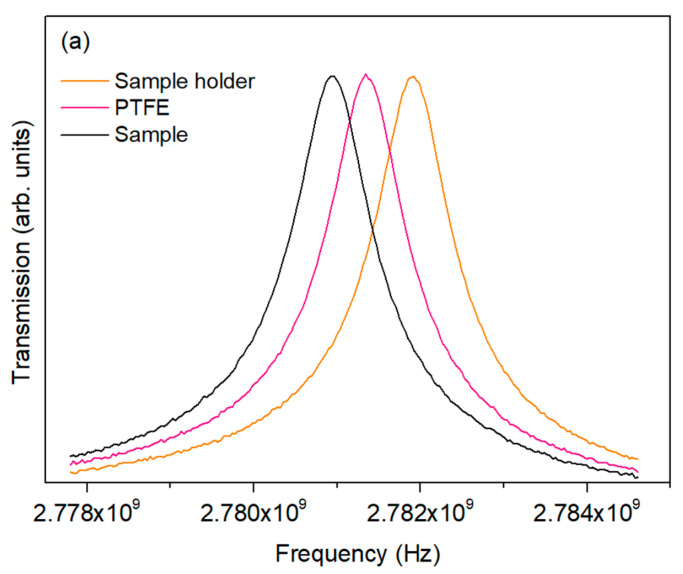

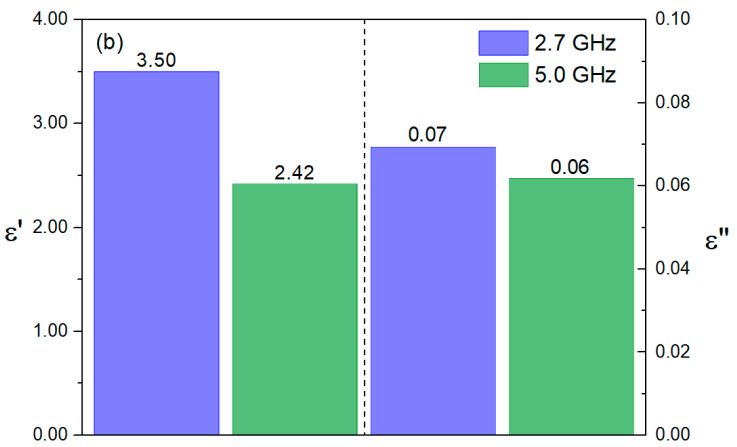
(**a**) Measured transmission spectra of the 2.7 GHz resonant cavity for the empty cavity, cavity loaded with a PTFE reference, and cavity loaded with the Bi-based sample, illustrating the resonance frequency shift and peak broadening. (**b**) Calculated real (ε′) and imaginary (ε″) parts of the complex permittivity measured at 2.7 and 5.0 GHz at room temperature using the *cavity resonant method* and the small perturbation theory.

In Figure 6a is presented the measured transmission of the 2.7 GHz cavity for the cavity with the sample holder, the sample holder filled with PTFE, and the sample holder filled with the sample, where the shift in the resonant frequency of the cavity, Δf, is observed. This shift shows that the sample exhibits a higher dielectric constant than PTFE, with peak broadening indicative of finite dielectric loss, consistent with standard microwave permittivity analysis. Figure 6b shows the calculated dielectric constant and dielectric loss measured at 2.7 and 5.0 GHz, at room temperature. Analyzing the results, it is evident that the sample exhibits a higher dielectric constant at 2.7 GHz compared to 5.0 GHz, while the dielectric loss remains low across both frequencies, indicating that the material is relatively stable and exhibits limited energy dissipation in the microwave range. Nevertheless, mixed valence states associated with Fe species in the Bi–Fe–Nb–O system may play a role in the microwave dielectric response and partially account for the moderate dielectric quality observed in the microwave frequency range.

### 3.4. Dielectric Characterization by Impedance Spectroscopy in the RF Range

The dielectric behavior of materials provides critical insight into their electrical response under alternating current (AC) fields, particularly in the radio-frequency (RF) range. Impedance spectroscopy is a powerful tool to investigate these properties, enabling the analysis of dielectric constant, dielectric loss, and relaxation phenomena across a broad frequency spectrum [24]. In this section, the dielectric response of the studied material is systematically investigated using multiple complementary approaches. Dielectric properties are first examined as a function of frequency to reveal polarization mechanisms and loss characteristics. Subsequently, electric modulus analysis is employed to elucidate relaxation dynamics and extract activation energies via the Arrhenius law. Finally, complex impedance analysis is carried out to understand conduction processes, quantify activation energies, and model the material’s behavior using an equivalent electrical circuit. Together, these analyses provide a comprehensive understanding of the material’s dielectric performance in the RF regime.

#### 3.4.1. Dielectric Properties

The dielectric constant, *ε*′; the dielectric loss, *ε*″; and the loss tangent, tan *δ*, were calculated using the following relations:

(3) $$\varepsilon^{\prime }=\frac{d}{A}\frac{C_{p}}{\varepsilon_{0}}$$

(4) $$\varepsilon^{\prime \prime }=\frac{d}{A}\frac{1}{{\omega \;R_{p}\;\varepsilon }_{0}}$$

(5) $$\tan\delta =\frac{\varepsilon^{\prime \prime }}{\varepsilon^{\prime }}$$

Here, *Cp* and *Rp* correspond to the experimentally determined capacitance and resistance, respectively; *ω* denotes the angular frequency; *d* is the thickness of the sample; *A* the electrode area; and *ε*_0_ the permittivity of free space (8.8542 × 10^−12^ F/m).

**Figure 7:**
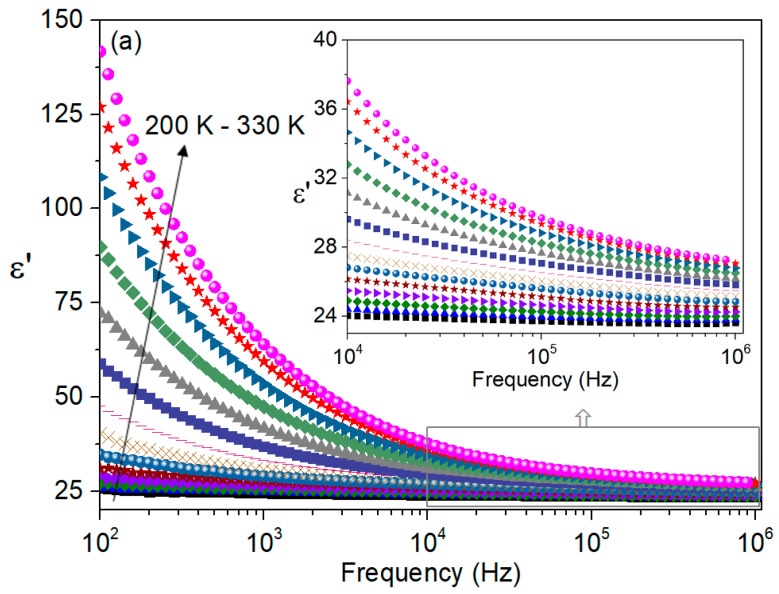

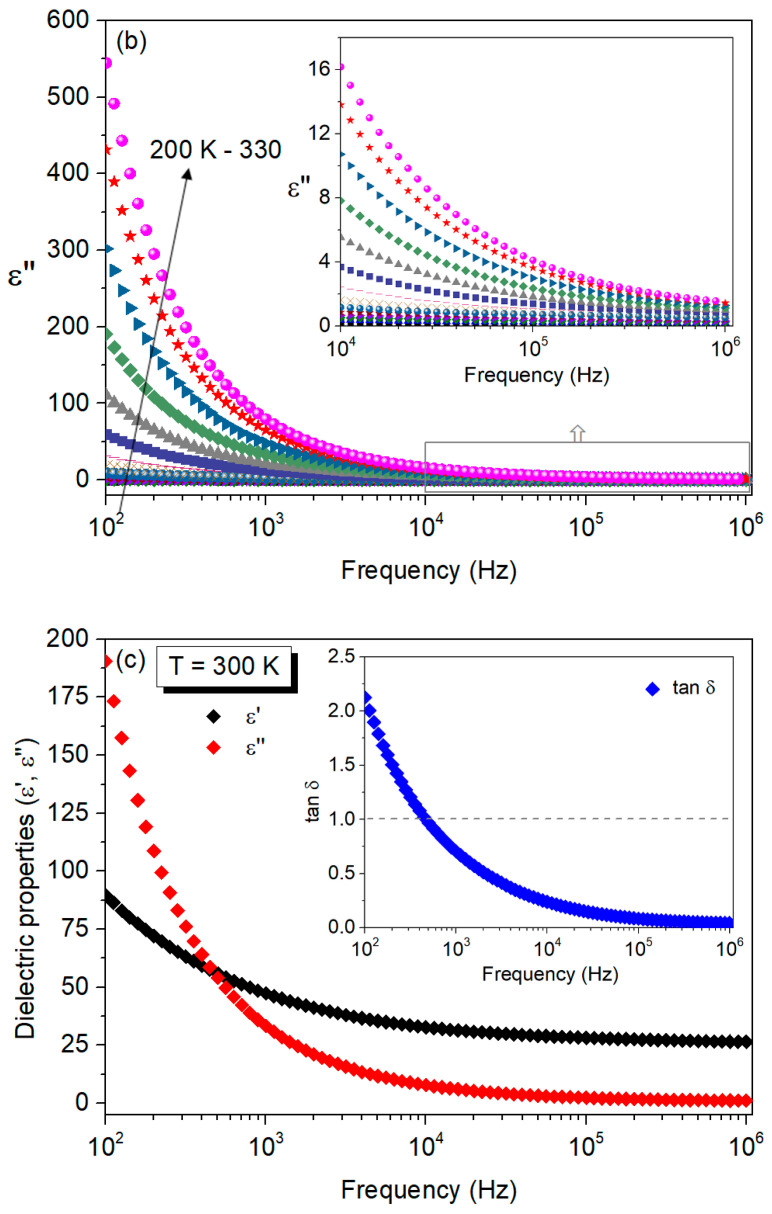
Frequency dependence of (**a**) the dielectric constant (ε′) and (**b**) dielectric loss (ε″) of the Bi-based system, measured by impedance spectroscopy for temperatures between 200 K and 330 K, in steps of 10 K. (**c**) Comparison of ε′ and ε″ at 300 K (inset: shows the corresponding frequency dependence of the loss tangent (tan δ), highlighting the relative balance between energy storage and dissipation).

Figure 7a shows the variation in the dielectric constant as a function of frequency and temperature for the bismuth-based sample. In the low-frequency region, it can be seen that *ε*′ decreases more sharply, whereas at higher frequencies the decrease becomes more gradual, eventually tending toward a nearly frequency-independent response. This type of dielectric dispersion is consistent with the Maxwell–Wagner interfacial polarization model and with Koop’s theory. Within this framework, the dielectric medium can be described as comprising highly conducting grain regions separated by less conductive grain-boundary layers, where grain-boundary contributions dominate the dielectric response at lower frequencies, while the intrinsic response of the grains becomes more prominent at higher frequencies [25].

The high dielectric constant is primarily attributed to space-charge polarization occurring at the grain boundaries. This polarization mechanism is associated with electron hopping between ions of the same element that exist in multiple valence states and are randomly distributed over crystallographically equivalent lattice sites. During this process, charge carriers must cross both the grains and the grain boundaries of the dielectric medium [14,26].

At low frequencies, the comparatively high resistance of the grain boundaries hinders charge transport, leading to the accumulation of charges at these interfaces and the development of pronounced space-charge polarization. As a result, the dielectric constant exhibits high values in this frequency range. With increasing frequency, the contribution from grain boundaries becomes less effective, and the dielectric constant decreases [14,26].

At higher frequencies, the dielectric response is increasingly governed by the grains themselves. However, the hopping motion of charge carriers is unable to follow the rapidly oscillating electric field, which further suppresses polarization. In general, polarizable species tend to lag behind the applied field at high frequencies, leading to a reduction in both the dielectric constant and its frequency dependence [14,26].

It is also observed that, at a fixed frequency, the dielectric constant increases with increasing temperature. As the temperature rises, lattice vibrations facilitate electron hopping, leading to an enhancement of ε′. The concentration of charge carriers increases with temperature and their accumulation at grain boundaries strengthens both interfacial and space-charge polarization. As a result, higher ε′ values are obtained at elevated temperatures [26].

The frequency dependence of the dielectric loss is depicted in Figure 7b. The higher values of ε″ at lower frequencies can be attributed mainly to conduction contributions. With increasing frequency, these conduction losses decrease, leading to a significant reduction in ε″ at higher frequencies [26]. Importantly, no clear relaxation peak is detected within the frequency range investigated.

Figure 7c compares *ε*′ and *ε*″ at room temperature (300 K). The inset shows the corresponding variation in tan *δ* with frequency. Except in the low-frequency region, tan *δ* remains below unity, implying that energy dissipation is consistently lower than energy storage [14]. This feature highlights the suitability of the material for energy-storage applications. A similar trend, dielectric loss being smaller than the dielectric constant at higher frequencies, can also be discerned from the insets of Figure 7a,b.

#### 3.4.2. Electric Modulus Analysis

The real, *M’*, and imaginary, *M*″, parts of the electric modulus, M*, are related to the permittivity by the following equations [27]:


(6) $$M^{\prime }=\;\frac{\varepsilon^{\prime }}{{\varepsilon^{\prime }}^{2}+{\varepsilon^{\prime \prime }}^{2}}$$

(7) $$M^{\prime \prime }=\;\frac{\varepsilon^{\prime \prime }}{{\varepsilon^{\prime }}^{2}+{\varepsilon^{\prime \prime }}^{2}}$$

One of the advantages of employing the modulus formalism to examine the dielectric response is that the strong fluctuations in permittivity and conductivity observed at low frequencies are suppressed. As a result, the usual limitations associated with electrode type and interface, space-charge injection, and conduction due to absorbed impurities, which may mask the relaxation mechanism in the permittivity representation, can be avoided. In addition, the impact of electrode polarization on the modulus spectra is diminished, especially when a good ohmic contact between the electrodes and the specimen is guaranteed, for instance by applying silver paint contacts [27,28].

**Figure 8:**
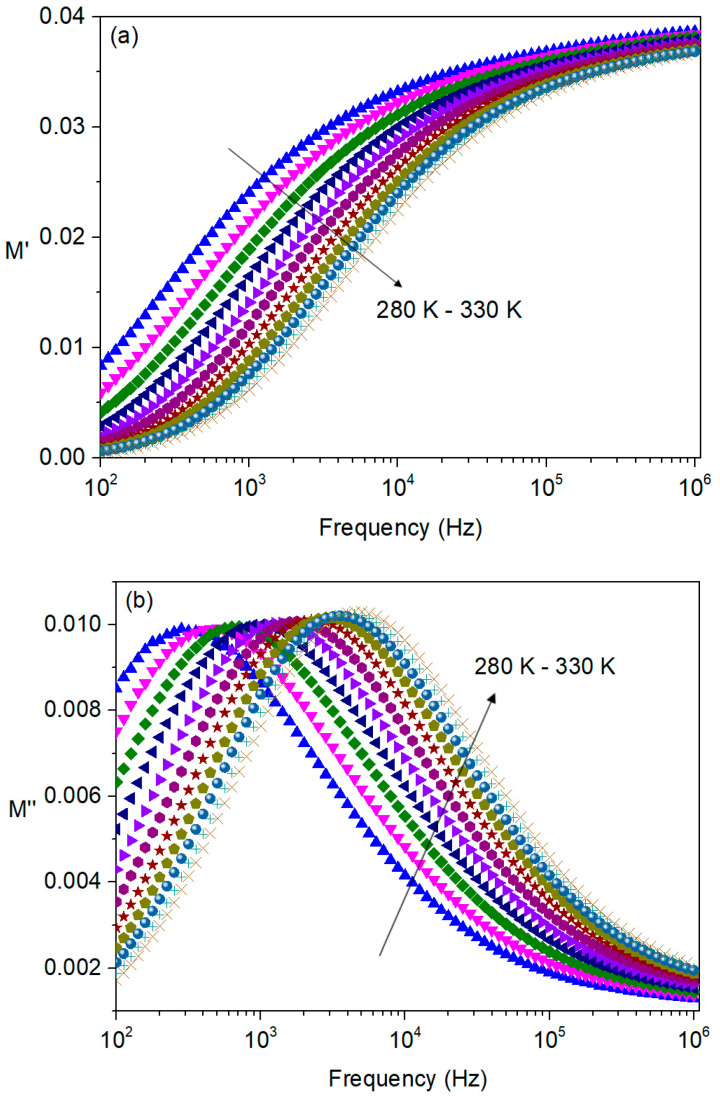

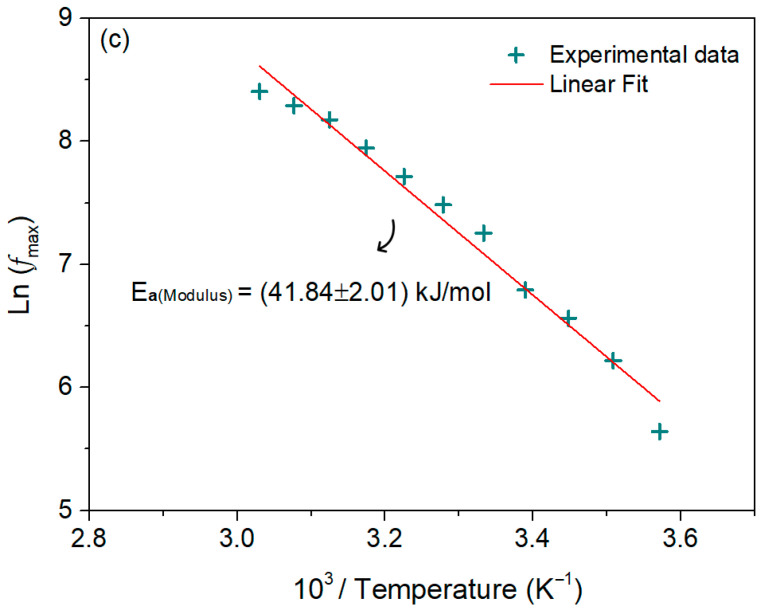
Frequency dependence of (**a**) the real part (M′) and (**b**) imaginary part (M″) of the electric modulus for temperatures between 280 K and 330 K, in steps of 5 K. The temperature-dependent shift in the M″ peak indicates a thermally activated relaxation process. (**c**) Arrhenius plot of the relaxation peak frequencies extracted from M″, used to determine the activation energy of the relaxation mechanism.

Figure 8a,b show the real and imaginary parts of the modulus, respectively, as a function of frequency and temperature.

As the frequency increases, *M*′ increases monotonically, making the frequency dependence less pronounced in the high-frequency region. In turn, *M*″ exhibits a clear peak, indicating the presence of a relaxation mechanism within the frequency range under investigation. It can be observed that the peak shifts to a higher frequency with the increase in temperature, indicating that the response of the dipoles to the external electric field becomes easier at higher temperatures.

Since the above-mentioned relaxation process is thermally activated, the activation energy, *E*_a_, can be calculated from the values of the maximum frequencies (*f*_max_) in each curve of *M*″(*f*) as a function of the temperature by fitting the experimental data to an Arrhenius equation [29]:

(8) $$f_{max}=\;f_{0}e^{\frac{-E_{a}}{k_{B}T}}$$

where *f*_max_ is the peak frequency at temperature *T*, *f*_0_ is a pre-exponential factor and *k_B_* is the Boltzmann constant. The logarithmic representation of the maximum frequency versus the inverse temperature, shown in Figure 7c, allows the calculation of the activation energy.

#### 3.4.3. Complex Impedance Analysis

The real and imaginary parts of the complex impedance, Z*, can be calculated from *ε*′ and *ε*″ using Equations (9) and (10) [29]:


(9) $$Z^{\prime \prime }=\;\frac{\varepsilon^{\prime \prime }d}{\omega A\varepsilon_{0}({\varepsilon^{\prime }}^{2}+{\varepsilon \prime \prime }^{2})}$$

(10) $$Z^{\prime }=\;\frac{\varepsilon^{\prime }d}{\omega A\varepsilon_{0}({\varepsilon^{\prime }}^{2}+{\varepsilon \prime \prime }^{2})}$$

**Figure 9:**
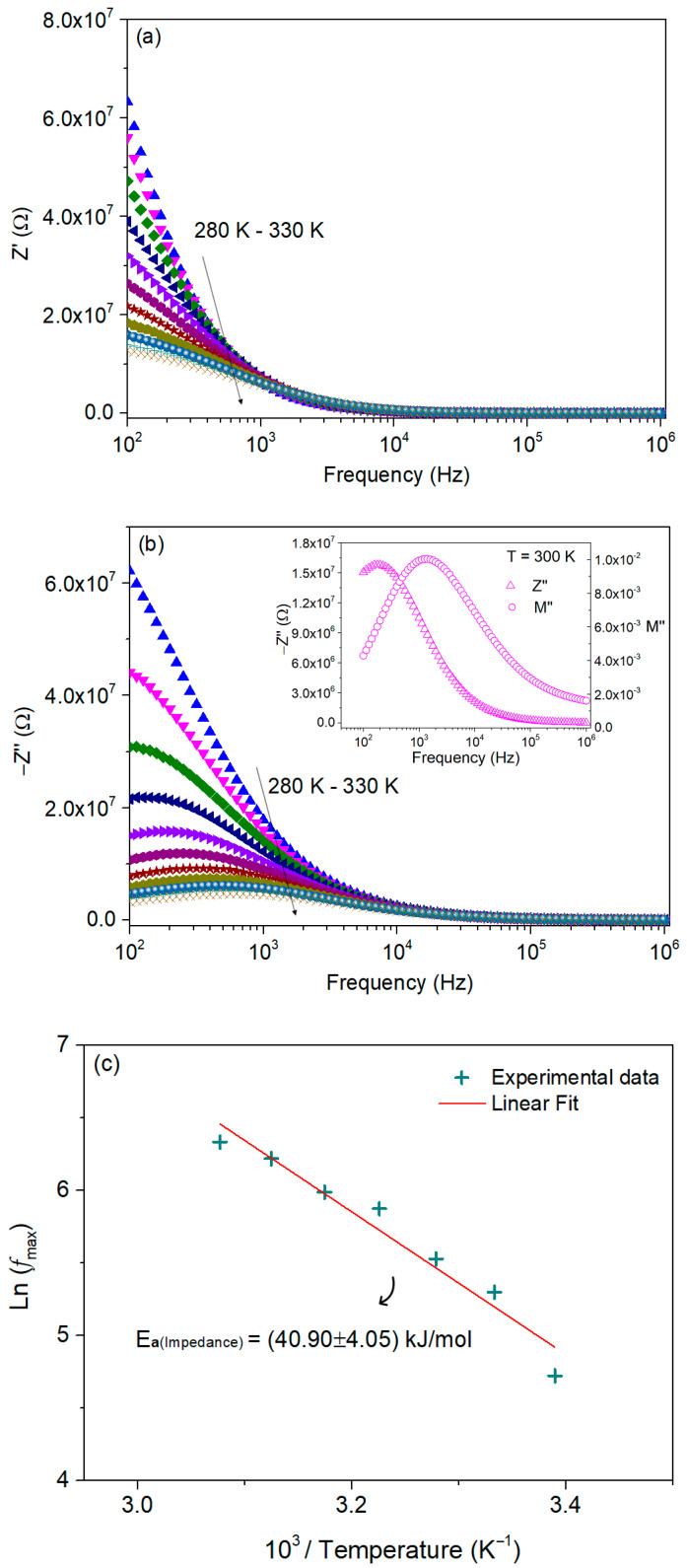

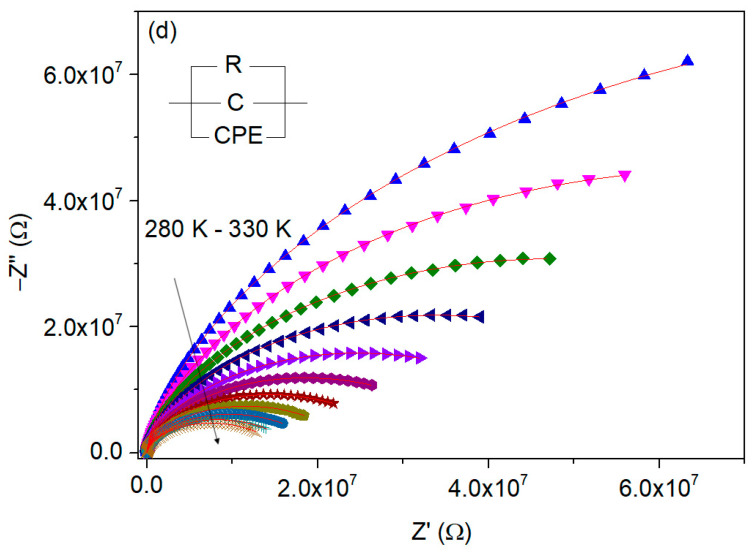
Frequency dependence of (**a**) the real (Z′) and (**b**) imaginary (Z″) parts of the complex impedance for temperatures between 280 K and 330 K, in steps of 5 K. Inset in (**b**) compares M″ and Z″ at 300 K to distinguish short-range and long-range charge transport contributions. (**c**) Arrhenius plot of the relaxation peak frequencies derived from the impedance response. (**d**) Nyquist plots (symbols) with corresponding equivalent-circuit fits (solid lines) at different temperatures (inset: shows the equivalent circuit model used for fitting).

Figure 9a,b show the real, *Z*′, and imaginary, *Z*″, parts of the impedance, respectively, as a function of frequency and temperature.

The real part of impedance Z′ decreases with both frequency and temperature. At low frequencies, Z′ exhibits high values due to charge accumulation and interfacial polarization effects, whereas at higher frequencies it becomes nearly constant and the curves for different temperatures merge. This merging behavior signifies that, at high frequencies, the response is dominated by bulk conduction processes that are largely temperature-independent. The overall decrease in Z′ with increasing temperature indicates thermally activated charge transport, consistent with a negative temperature coefficient of resistance behavior [15,30].

The variation in the imaginary part of impedance, *Z*″, with frequency exhibits, for temperatures above 300 K, broad relaxation peaks whose position and intensity depend on temperature. The shift in the peak position toward higher frequencies with increasing temperature indicates a decrease in the relaxation time, suggesting that the relaxation process is thermally activated. A progressive decrease in the peak height with increasing temperature and the convergence of all spectra at higher frequencies can also be observed. The reduction in peak amplitude implies enhanced charge carrier mobility and a weakening of polarization effects at elevated temperatures. Once again, the merging of spectra at high frequencies supports that the impedance response is dominated by bulk conduction mechanisms that are nearly temperature-independent [31].

Figure 9c shows the logarithmic representation of the maximum frequency versus the inverse temperature and the associated activation energy calculated from Equation (8).

It can be seen that the activation energy calculated from the modulus formalism for the relaxation mechanisms, 41.84 ± 2.01 kJ/mol, shows a remarkable similarity to the one obtained through the impedance spectra, 40.90 ± 4.05 kJ/mol. Such consistency strongly indicates that both approaches describe the same relaxation mechanism, reflecting a single underlying physical process [32].

A comparative plot of the imaginary parts of the modulus and impedance as a function of frequency, shown in the inset of Figure 9, is a useful tool for identifying the response associated with the smallest capacitances and the highest resistances [33]. This representation helps clarify whether the observed relaxation originates from long-range or short-range motion of charge carriers. According to previous reports, when the maxima of Z′′ and M′′ occur at the same frequency, the relaxation is attributed to long-range charge transport; when the peak positions differ, the mechanism is associated with short-range carrier motion. In the present case, the peaks do not coincide, indicating that the relaxation arises from short-range charge-carrier dynamics [28].

Figure 9d presents the corresponding Nyquist plot, where the symbols represent the experimental data, and the solid red line corresponds to the simulated response obtained for the equivalent circuit chosen as a fitting model. The model, also depicted in Figure 9d, consists of a Constant Phase Element (CPE) in parallel with a resistor (R) and a capacitor (C), representing the non-ideal capacitive behavior observed in the impedance spectrum.

The observed semicircular shape is characteristic of a charge transfer resistance coupled with double-layer capacitance behavior [34]. The fitting was carried out using EIS Spectrum Analyzer Software, version 1.0, and the excellent agreement between the experimental data and the simulated curve suggests that the selected equivalent circuit reliably represents the electrical behavior of the system.

Table 1 summarizes the estimated circuit parameter values obtained by fitting the experimental data using the mentioned simulation tool, considering that in the frequency domain, the impedance of a constant phase element is given by [35,36]:


(11) $$Z=Af^{-n}\left(\cos\left(\frac{n\pi }{2}\right)-j\;sin\left(\frac{n\pi }{2}\right)\right)$$

Here, A represents the impedance parameter evaluated at an angular frequency of 1 rad/s; f denotes the frequency; and *n* is the exponent, which equals 1 for an ideal capacitive response. The corresponding admittance coefficient is given by Q = 1/A.

The parameters extracted from the fit, summarized in Table 1, show that the capacitance remains relatively constant (~5.3–5.5 pF) across the temperature range, suggesting stable dielectric storage. Meanwhile, the resistance decreases sharply with increasing temperature (from 250 MΩ at 280 K to 17.1 MΩ at 330 K), confirming thermally activated conduction. The CPE parameters (*Q* and *n*) reveal non-ideal capacitive behavior, with *Q* increasing from 0.203 ns to 0.714 ns and *n* remaining near 0.53, reflecting distributed relaxation processes and significant deviations from ideal Debye behavior. These results confirm that the electrical properties of the system are temperature-dependent while maintaining stable dielectric performance.

To place the dielectric response of the present Bi–Fe–Nb oxide system in context, Table 2 compares representative dielectric properties reported at 1 MHz for ceramic materials processed over a wide range of temperatures and proposed for LTCC-related applications. The selected examples span different microstructural states and dielectric performance levels, illustrating the trade-off between processing temperature, densification, and dielectric behavior. An analysis of Table 2 shows that materials sintered at moderate to high temperatures (≥1000 °C) typically exhibit higher permittivity and lower dielectric loss, reflecting enhanced densification and reduced defect concentrations. In contrast, ceramics processed at lower temperatures generally display reduced permittivity and increased dielectric loss, commonly associated with higher porosity, residual amorphous phases, and stronger interfacial polarization effects. Within this framework, the present Bi–Fe–Nb oxide system exhibits moderate dielectric parameters at 1 MHz but is distinguished by achieving a stable dielectric response at an exceptionally low processing temperature. These observations underscore the inherent trade-off between processing temperature, microstructural state, and dielectric performance, emphasizing that the primary contribution of this work lies in ultra-low-temperature processability rather than in maximizing dielectric performance.

## 4. Conclusions

A multicomponent Bi–Fe–Nb oxide system was synthesized by a modified citrate sol–gel route and thermally treated at only 400 °C, enabling the investigation of its early-stage structural evolution and dielectric response. Structural characterization revealed a two-phase system composed of crystalline BiOCl and a partially amorphous Bi–Fe–Nb–O matrix, reflecting the complex crystallization kinetics of bismuth-containing multicomponent oxides at low temperatures. DLS measurements further supported this microstructural complexity by revealing strongly polydisperse particle populations, characterized by a hydrodynamic Z-average of (640 ± 7) nm and the predominance of micrometer-scale aggregates. The emergence of multimodal distributions, with major modes near 1.36 µm and 1.42 µm, and only a residual nanoscale fraction, indicates extensive aggregation and low colloidal stability, consistent with the heterogeneous morphology observed by SEM. Microwave measurements revealed stable dielectric losses and permittivity across the investigated frequencies, although the overall microwave dielectric performance remains moderate. These results nevertheless provide a basis for further studies aimed at optimization or the exploration of alternative functional applications. Impedance spectroscopy in the RF range demonstrated strong interfacial polarization at low frequencies, thermally activated charge transport, and a relaxation process whose activation energies extracted from the modulus and impedance formalisms were in close agreement, indicating a common underlying mechanism. Equivalent-circuit modeling using a CPE–R–C configuration reproduced the experimental data accurately, revealing non-Debye relaxation behavior with an almost constant capacitance and a pronounced temperature dependence of resistance. Overall, these results show that the Bi–Fe–Nb system retains stable dielectric storage capabilities while exhibiting characteristic thermally activated electrical behavior, highlighting its potential for incorporation into future LTCC and RF dielectric components processed at ultra-low temperatures.

## Figures and Tables

**Table 1:** Fitted parameters for the electrical parameters obtained from the equivalent circuit model used to describe the sample’s impedance behavior.

T (K)	C (pF ± %)	R (MΩ ± %)	Q (ns ± %)	n (a.u. ± %)
280	5.48 ± 0.17	250 ± 0.27	0.204 ± 0.14	0.526 ± 0.04
285	5.30 ± 0.21	155 ± 0.22	0.203 ± 0.15	0.552 ± 0.04
290	5.34 ± 0.24	108 ± 0.20	0.264 ± 0.15	0.541 ± 0.04
295	5.34 ± 0.21	77.1 ± 0.14	0.314 ± 0.12	0.538 ± 0.03
300	5.28 ± 0.23	55.8 ± 0.12	0.360 ± 0.12	0.540 ± 0.03
305	5.40 ± 0.26	42.5 ± 0.12	0.442 ± 0.13	0.530 ± 0.03
310	5.45 ± 0.32	33.0 ± 0.13	0.508 ± 0.16	0.527 ± 0.04
315	5.32 ± 0.44	26.3 ± 0.15	0.533 ± 0.21	0.534 ± 0.05
320	5.29 ± 0.26	22.0 ± 0.08	0.605 ± 0.12	0.531 ± 0.03
325	5.26 ± 0.32	18.9 ± 0.10	0.650 ± 0.14	0.531 ± 0.03
330	5.30 ± 0.47	17.1 ± 0.14	0.714 ± 0.21	0.526 ± 0.05

**Table 2:** Comparison of dielectric properties at 1 MHz and room temperature and corresponding processing/sintering conditions for representative ceramic dielectrics processed at low and moderate temperatures, including the present Bi–Fe–Nb oxide system, highlighting the balance between processing temperature and dielectric performance.

Material/System	Processing/Sintering Conditions (Temperature, Time)	ε′	ε″	tan δ	Reference
Bi–Fe–Nb oxide system	400 °C, 4 h	26.50	1.14	0.04	This work
Ni_0.37_Cu_0.20_Zn_0.43_-Fe_1.92_O_3.88_	900 °C, 2.5 h	15.99	----	0.019	[37]
Li_2_BaP_2_O_7_	775 °C, 4 h	≈7.1	≈0.22	≈0.03	[38]
Bi_12_SiO_20_	800 °C, 5 h	≈44.5	----	≈0.02	[39]
NiO-ZnO	500 °C, 4 h	≈4.2	----	≈0.07	[40]
(Pb_0.92_La_0.02_Sr_0.06_)[(Zr_0.5_Sn_0.5_)_0.9_Ti_0.1_]_0.995_O_3_ − 0.6%wt BASK − 0.4%wt Sm_2_O_3_	1040 °C, 2 h	≈415	----	≈0.01	[41]

## Data Availability

The data supporting the findings of this study are available from the corresponding author upon reasonable request.

## References

[1] Sebastian M.T., Wang H., Jantunen H. (2016). Low temperature co-fired ceramics with ultra-low sintering temperature: A review. Curr. Opin. Solid. State Mater. Sci..

[2] Fan L., Liao Y., Li Y., Li F., Li J., Chen W., Zhao Q. (2025). Low loss and excellent stability of Zn_0.7_Mg_0.3_TiO_3_ ceramics with V_2_O_5_–TiO_2_ addition for application in low-temperature co-fired ceramic technology. J. Mater. Chem. C Mater..

[3] Wang C.-H., Zhang K.-H., Wang W., Bao J., Jiang J.-P., Zhou K.-H., Li J., Liang C., Liu D.-W., Darwish M.A. (2025). A comprehensive study on low-temperature sintering and microwave/terahertz dielectric properties of BaO–P_2_O_5_ binary ceramics. J. Mater. Chem. C Mater..

[4] Ma X.-H., Qu Q., Wu H., Zhang Z., Ma X. (2024). Low-Temperature Sintering and Microwave Dielectric Properties of Cu_x_Zn_1−x_Ti_0.2_Zr_0.8_Nb_2_O_8_ Ceramics with the Aid of LiF. Materials.

[5] He G.-Q., Miao J., Wu F.-F., Wang W., Bao J., Jiang J.-P., Liu D.-W., Darwish M.A., Zhou T., Xu D.-M. (2025). Advancements in microwave dielectric ceramics with K20 for 5G/6G communication systems: A review. J. Mater. Chem. C Mater..

[6] Synkiewicz-Musialska B., Szwagierczak D. (2025). Impact of AlF_3_-CaB_4_O_7_ Doping on Terahertz Dielectric Properties and Feasibility of Low/Ultra-Low Temperature Co-Fired Ceramics. Materials.

[7] Althumairi N.A., Hjiri M., Aldukhayel A.M., Jbeli A., Nassar K.I. (2025). Recent Advances in Dielectric and Ferroelectric Behavior of Ceramic Nanocomposites: Structure Property Relationships and Processing Strategies. Nanomaterials.

[8] Wang D., Li L., Du M., Zhan Y. (2021). A low-sintering temperature microwave dielectric ceramic for 5G LTCC applications with ultralow loss. Ceram. Int..

[9] Wang F., Zhang W., Chen X., Mao H., Liu Z., Bai S. (2020). Low temperature sintering and characterization of La_2_O_3_-B_2_O_3_-CaO glass-ceramic/LaBO_3_ composites for LTCC application. J. Eur. Ceram. Soc..

[10] Wu F.-F., Zhou D., Xia S., Zhang L., Qiao F., Pang L.-X., Sun S.-K., Zhou T., Singh C., Sombra A.S.B. (2022). Low sintering temperature, temperature-stable scheelite structured Bi[V_1−x_(Fe_1/3_W_2/3_)_x_]O_4_ microwave dielectric ceramics. J. Eur. Ceram. Soc..

[11] Can F., Courtois X., Duprez D. (2021). Tungsten-Based Catalysts for Environmental Applications. Catalysts.

[12] Wang W., Wang S., Sun J., Wang Q., Fang B. (2023). Low-Temperature Sintering of Bi(Ni_0.5_Ti_0.5_)O_3_-BiFeO_3_-Pb(Zr_0.5_Ti_0.5_)O_3_ Ceramics and Their Performance. Materials.

[13] Xue X., Li X., Fu C., Zhang Y., Guo J., Wang H. (2023). Sintering characteristics, phase transitions, and microwave dielectric properties of low-firing [(Na_0.5_Bi_0.5_)_x_Bi_1−x_](W_x_V_1−x_)O_4_ solid solution ceramics. J. Adv. Ceram..

[14] Devesa S., Amorim C.O., Belo J.H., Araújo J.P., Teixeira S.S., Graça M.P.F., Costa L.C. (2024). Comprehensive Characterization of Bi_1.34_Fe_0.66_Nb_1.34_O_6.35_ Ceramics: Structural, Morphological, Electrical, and Magnetic Properties. Magnetochemistry.

[15] Devesa S., Graça M.P., Costa L.C. (2021). Impedance Spectroscopy Study of Bi_1.34_Fe_0.66_Nb_1.34_O_6.35_ Ceramics. J. Electron. Mater..

[16] Devesa S., Rooney A.P., Graça M.P., Cooper D., Costa L.C. (2021). Williamson-hall analysis in estimation of crystallite size and lattice strain in Bi_1.34_Fe_0.66_Nb_1.34_O_6.35_ prepared by the sol-gel method. Mater. Sci. Eng. B.

[17] Bannister F.A. (1934). Crystal structure of bismuth oxyhalides. Nature.

[18] Yang J., Xie T., Liu C., Xu L. (2018). Dy(III) Doped BiOCl Powder with Superior Highly Visible-Light-Driven Photocatalytic Activity for Rhodamine B Photodegradation. Nanomaterials.

[19] Wu S., Wang C., Cui Y. (2014). Controllable growth of BiOCl film with high percentage of exposed {001} facets. Appl. Surf. Sci..

[20] Bárdos E., Márta V.A., Fodor S., Kedves E.-Z., Hernadi K., Pap Z. (2021). Hydrothermal Crystallization of Bismuth Oxychlorides (BiOCl) Using Different Shape Control Reagents. Materials.

[21] André P.S., Costa L.C., Devesa S. (2004). Fabry-perot-based approach for the measurement of complex permittivity of samples inserted in resonant cavities. Microw. Opt. Technol. Lett..

[22] Walter S., Schwanzer P., Steiner C., Hagen G., Rabl H.P., Dietrich M., Moos R. (2022). Mixing rules for an exact determination of the dielectric properties of engine soot using the microwave cavity perturbation method and its application in gasoline particulate filters. Sensors.

[23] Mark J.E. (2009). Polymer Data Handbook.

[24] Kumari K., Kumar S., Sharma R.K., Kumar R., Vij A., Ahmed F., Kumar A., Hashim M., Koo B.H. (2023). Dielectric properties of spinel ferrite nanostructures. Ferrite Nanostructured Magnetic Materials.

[25] Neiva J., Benzarti Z., Carvalho S., Devesa S. (2024). Green Synthesis of CuO Nanoparticles—Structural, Morphological, and Dielectric Characterization. Materials.

[26] Sarkar R., Sarkar B., Pal S. (2021). Dielectric properties and thermally activated relaxation in monovalent (Li+1) doped multiferroic GdMnO_3_. Appl. Phys. A.

[27] Tian F., Ohki Y. (2014). Electric modulus powerful tool for analyzing dielectric behavior. IEEE Trans. Dielectr. Electr. Insul..

[28] Malleo D., Nevill J.T., Van Ooyen A., Schnakenberg U., Lee L.P.H. (2010). Morgan Characterization of electrode materials for dielectric spectroscopy. Rev. Sci. Instrum..

[29] Bhadauria P.P.S., Kolte J. (2022). Impedance and AC conductivity analysis of La-substituted 0.67BiFeO_3_–0.33BaTiO_3_ solid solution. Appl. Phys. A.

[30] Adak M.K., Dhak D. (2017). Density of states and impedance behaviour of transition metal substituted SrBi_2_Nb_2_O_9_ ferroelectric nanoceramics prepared by chemical process. J. Mater. Sci. Mater. Electron..

[31] Mohapatra S.R., Sahu B., Badapanda T., Pattanaik M.S., Kaushik S.D., Singh A.K. (2016). Optical, dielectric relaxation and conduction study of Bi_2_Fe_4_O_9_ ceramic. J. Mater. Sci. Mater. Electron..

[32] Marijan S., Pavić L. (2024). Solid-state impedance spectroscopy studies of dielectric properties and relaxation processes in Na_2_O-V_2_O_5_-Nb_2_O_5_-P_2_O_5_ glass system. Int. J. Miner. Metall. Mater..

[33] Wang S., Chen L. (2016). Interfacial transport in lithium-ion conductors. Chin. Phys. B.

[34] Toloman D., Gungor A., Popa A., Stefan M., Macavei S., Barbu-Tudoran L., Varadi A., Yildirim I.D., Suciu R., Nesterovschi I. (2025). Morphological impact on the supercapacitive performance of nanostructured ZnO electrodes. Ceram. Int..

[35] da Silva G.M.G., Faia P.M., Mendes S.R., Araújo E.S. (2024). A Review of Impedance Spectroscopy Technique: Applications, Modelling, and Case Study of Relative Humidity Sensors Development. Appl. Sci..

[36] Faia P.M., Ferreira A.J., Furtado C.S. (2009). Establishing and interpreting an electrical circuit representing a TiO_2_–WO_3_ series of humidity thick film sensors. Sens. Actuators B Chem..

[37] Ling W., Zhang H., Song Y., Liu Y., Li Y., Su H. (2009). Low-Temperature Sintering and Electromagnetic Properties of Ferroelectric–Ferromagnetic Composites. J. Magn. Magn. Mater..

[38] Xu X., Du W., Xu K., Chen S., Wu H., Shan L. (2025). Optical, Electrical and Microwave/Terahertz Dielectric Properties of Novel Low-Temperature Sintered Quaternary-Phase Li_2_O-BaO-P_2_O_5_ Pyrophosphate Ceramic for Patch Antenna Application. Ceram. Int..

[39] Du J., Feng Q., Jia R., Hu S., Fujita T., Luo N., You H., Chen X., Cen Z., Yuan C. (2025). A Low-Temperature-Sintering Bi_12_SiO_20_ Ceramics with Ultrahigh Energy Efficiency and Breakdown Strength of ~1.05MV/Cm. J. Alloys Compd..

[40] Rafi M., Bin Khatab Abbasi B., Ahmad S., Abd EL-Gawaad N.S. (2025). Temperature Dependent Charge Transport and Conduction Mechanism through Different Electroactive Regions in NiO-ZnO Heterostructure Nanocomposite by Using Impedance Spectroscopy. Ceram. Int..

[41] Wang X., Zhao H., Tang M., Wang G., Xu R., Feng Y., Li Z., Wei X., Xu Z. (2024). Samarium-Modified PLZST-Based Antiferroelectric Energy Storage Ceramics for Low-Temperature Sintering in Reducing Atmosphere. Ceram. Int..

